# An Evaluation of Commercial Fluorescent Bead-Based Luminex Cytokine Assays

**DOI:** 10.1371/journal.pone.0002535

**Published:** 2008-07-02

**Authors:** Joel Fleury Djoba Siawaya, Teri Roberts, Chantal Babb, Gillian Black, Hawa Jande Golakai, Kim Stanley, Nchinya Bennedict Bapela, Eileen Hoal, Shreemanta Parida, Paul van Helden, Gerhard Walzl

**Affiliations:** 1 Department of Biomedical Sciences, Division of Molecular Biology and Human Genetics, Stellenbosch University Cape-Town, Stellenbosch, South Africa; 2 Department of Immunology, Max Planck Institute for Infection Biology, Berlin, Germany; University of Bremen, Germany

## Abstract

The recent introduction of fluorescent bead-based technology, allowing the measurement of multiples analytes in a single 25–50 µl sample has revolutionized the study of cytokine responses. However, such multiplex approaches may compromise the ability of these assays to accurately measure actual cytokine levels. This study evaluates the performance of three commercially available multiplex cytokine fluorescent bead-based immunoassays (Bio-Rad's Cytokine 17-plex kit; LINCO Inc's 29-plex kit; and RnD System's Fluorokine-Multi Analyte Profiling (MAP) base kit A and B). The LINCO Inc kit was found to be the most sensitive assay for measuring concentrations of multiple recombinant cytokines in samples that had been spiked with serial dilutions of the standard provided by the manufacturer, followed respectively by the RnD Fluorokine-(MAP) and Bio-Rad 17-plex kits. A positive correlation was found in the levels of IFN-γ measured in antigen stimulated whole blood culture supernatants by the LINCO Inc 29-plex, RnD Fluorokine-(MAP) and RnD system IFN-γ Quantikine ELISA kits across a panel of controls and stimulated samples. Researchers should take the limitation of such multiplexed assays into account when planning experiments and the most appropriate use for these tests may currently be as screening tools for the selection of promising markers for analysis by more sensitive techniques.

## Introduction

Cytokines are important modulators of immune response pathways [7], [18, 19]. Cytokine expression profiling (CEP) has become a popular and established method for the identification and characterisation of disease-associated immune responses [4], [6], [8], [9]. Previously, CEP was a laborious process requiring substantial sample volumes when multiple cytokines were under investigation. However, CEP methodology has been revolutionised by the recent introduction of fluorescent bead-based luminex technology, a capture/detection sandwich type immunoassay allowing the measurement of up to 100 different analytes in a single 50 µl sample [16].

The reduced sample volume and time-saving advantages of the luminex system have made it an attractive method for large-scale cross-sectional, association or cohort studies which investigate the host immune response [1], [13], [15], [16]. Khan *et al*. [11] have comparatively assessed multiplex kits from LINCO Research, Bio-Rad Laboratories, RnD Systems and Biosource International and compared them to an enzyme-linked immunosorbant assay (ELISA). The comparison was based on the measurement of a sample of five cytokines (serum samples from healthy individuals intravenously injected with endotoxin). They reported that the cytokine concentrations, as measured by the different kits, showed similar trends, although the absolute concentrations measured were different.

There are also a number of reports validating luminex systems. These studies often used kits with a narrow panel of cytokines. The present study not only has the advantage of combining a head-to-head comparison of different kits and assays on their ability to measure cytokine levels in blood samples, but is also the first independent study to comparatively assess the recovery of each cytokine by the commercially available Bio-Rad 17-plex, LINCO 29-plex and RnD Fluorokine-(MAP) base kits A and B (13 cytokines total) with reference to instrument settings and calibration.

## Methods

### Study design

This study followed an integrated methodology, comparing 3 commercially available multi-plex luminex kits (Cytokine 17-plex kit by Bio-Rad; a 29-plex kit by LINCO Research; and Fluorokine-Multi Analytes Profiling (MAP) kit) by RnD System as well as the RnD Systems IFN-γ Quantikine ELISA kit. We have used the following two approaches: 1) Measurement of recombinant cytokines in serum and in unstimulated diluted whole blood culture supernatant samples, each spiked with serial dilutions of the multiplex standard provided by the luminex kit manufacturer in order to calculate the recovery (accuracy) of the assay for each of the different cytokines; 2) Measurement of native, induced IFN-γ, *in vitro*, whole blood culture supernatants where, whole blood culture supernatants were stimulated with *Mycobacterium tuberculosis *(Mtb) antigens or Bacille Calmette Guerin (BCG).

### Definitions

#### Recovery

Ratio of the observed amount of cytokine compared to the expected known amount of cytokine in a sample, expressed as a percentage. An acceptable recovery falls within the range of 70–130% *(Bio-Rad Principles of Curve Fitting for Multiplex Sandwich Immunoassays, Rev B*).

The following formula was used to calculate recovery:
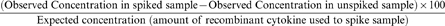
The definition and calculations of recovery were obtained from the RnD-Systems spike and recovery immunoassay sample validation protocol.

#### Reading

Reported fluorescence of the sample

#### Positive reading

Reported fluorescence of a sample that is above background fluorescence and corresponds to a positive concentration.

#### RP1

RP1 represents the fluorescence channel used for assay quantification. Low RP1 is the fluorescent channel recommended for quantification of a wide range of cytokines with a wide dynamic range of concentrations; whereas high RP1 is recommended for quantification of low concentrations of cytokines as it provides greater sensitivity.

#### 5 PL-(parameters logistic) Regression Curve

A standard curve build upon a five parameters logistic equation and that corrects for asymmetry in the curve shape.

#### Manufacturer 1

Bio-Rad

#### Manufacturer 2

LINCO Research

#### Manufacturer 3

RnD Systems

### Manufacture 1 assay

#### Experiment 1

The Bio-Rad human cytokine 17-plex assay was carried out according to the manufacturer's instructions, with a few exceptions as stipulated below. Briefly, a nine-point standard curve was generated by performing serial dilutions of the reconstituted normalised standard (lot # 5004060). This was not reconstituted with standard diluent, but rather with unstimulated whole blood assay supernatant, which was prepared by diluting whole blood one in ten with RPMI-1640 (GIBCO) and incubating at 37°C, 5% CO_2_ for six days. This was done in order to ensure that the matrix used in the generation of the standard curve resembled that of the samples as closely as possible as preliminary test showed that this method was superior to dilution of standards in standard diluent (data not shown). In order to assess recovery, supernatant samples SN1, SN2 and SN3 were each spiked at three concentrations (ranging from 7–5461 pg/ml) with recombinant cytokine using the Bio-plex kit standard. The assays were run in duplicate, which produced in total six concentration replicates. In order to keep the matrix of the spiked samples as similar as possible to the matrix of the standard curve, the volume of reconstituted standard used to spike the samples in all experiments was kept to a minimum and never exceeded 10 µl. A 50 µl volume of each sample, control or standard was added to a 96 well plate (provided with the kit) containing 50 µl of antibody coated fluorescent beads. Biotinylated secondary and streptavidin-PE antibodies were added to the plate with alternate incubation and washing steps. After the last wash step, 125 µl of wash buffer was added to the wells, the plate incubated and read on the Bio-plex array reader, using a 5 PL regression curve to plot the standard curve. Samples and controls were read at both a low RP1 target setting (used to maximize assay sensitivity when the expected concentrations are below 3 200 pg/ml) and a high RP1 target setting (used for broad range concentrations) on the Bio-plex suspension array using a high throughput fluidics (HTF) system (cat# 171000005). Data was subsequently analysed using the Bio-plex manager software, version 3.

#### Experiment 2

The standard curve was generated and the assay conducted as described above in the section on the *Bio-Rad human 17-plex assay experiment 1*. Two sets of samples were used. The first set was generated using whole blood from a healthy laboratory donor diluted one in ten with RPMI-1640 with glutamax and stimulated with different Mtb antigens, (generously donated by Tom Ottenhoff, Leiden University), and a phytohaemagglutinin (PHA)-stimulated positive control. Unstimulated culture supernatant served as a negative control. The second set of samples was generated from unstimulated whole blood culture as described above. Supernatants were harvested on day seven and spiked at five different concentrations with recombinant cytokine from the Bio-Rad standard (lot # 5004060. The six concentrations at which samples were spiked were unique for each cytokine with the lowest spike ranging from 2–43 pg/ml and the highest from 1191–8062 pg/ml). The results of these tests were used to calculate recovery.

### Manufacturer 2 assay

#### Experiment 1

A human 29-plex LINCO assay (cat no HCYTO -60-K-PMX29) was done according to manufacturer's instructions. Briefly, a standard curve ranging from 3.2 pg/ml to 10 000 pg/ml was generated by serial dilution of reconstituted standard. Two sets of samples were used, as described earlier, with the exception that for the second set of samples the LINCO Research standard (provided with the kit) was used to spike unstimulated whole blood culture at final concentrations of 5000, 1000, 500, 50 and 10 pg/ml. Additionally for the assessment of the LINCO kit reproducibility five aliquots of the same PHA-stimulated whole blood supernatant were produced and each aliquot was run in five different experiments on different days. Briefly the filter plates were blocked by pipetting 200 µl of assay buffer into each well. After 10 minutes the assay buffer was discarded by vacuum aspiration and 25 µl of assay diluent was added to the wells designated for the samples, while 25 µl of RPMI-1640 with glutamax (GIBCO) was added to the wells designated for standards. According to the plate layout, 25 µl of either standard or sample was then added to the appropriate wells after which 25 µl of antibody coated fluorescent beads was added. Biotinylated secondary (detection) and Streptavidin-PE-labelled antibodies were then added to the plate respectively, with alternate incubation and washing steps. Finally 100 µl of sheath fluid was added to the wells and the plate read immediately on the Bio-plex array reader, at high and low RP1 targets, using a 5 PL regression curve.

#### Experiment 2

A repeat of the experiment 1 was done by measuring 21 cytokines in two sets of samples including 171 Mtb antigens stimulated culture supernatant and spiked unstimulated whole blood culture (as previously described). Only this time samples were spiked at 2 different concentrations. The Plate was read at low RP1 targets as previously described.

### Manufacturer 3 assay

#### Experiment 1

The assay was done according to the manufacturer's instructions. Briefly, the standard curves for the RnD System fluorokine-(MAP) human base kits A (cat # LUH000) and B (cat # LUH001) were generated by reconstitution of standards in standard diluent provided with the kit. Samples included the same set of antigen-stimulated whole blood culture supernatants used for the Bio-plex experiment 2 and LINCO 29-plex assays described earlier, as well as serum (diluted one in four) and whole blood supernatant spiked at six different concentrations with recombinant cytokine from the RnD System's standard (Part # 895531, lot # 238222 and Part # 895546, lot # 238223 [base kit A] and Part # 892794, lot # 233020 [base kit B]). The six concentrations at which samples were spiked were unique for each cytokine with the lowest spike ranging from 14–600 pg/ml and the highest from 950–19 000 pg/ml. An eight-point standard curve, with each cytokine spanning its own unique specific range, was generated and 50 µl of each standard and sample were added to a 96-well plate containing fluorescent antibody coated beads. After alternate incubation and washing steps, detection and PE-labelled secondary antibodies were added and the plate read on the Bio-plex array reader, at a low RP1 target, using a 5 PL regression curve.

#### Experiment 2

The assay was done according to the manufacturer's instructions. Serum (diluted one in four) and whole blood culture supernatant were spiked with seven different concentrations (ranging from 5–1700 pg/ml) of recombinant cytokine from the RnD System's standard and measured as previously described.

### RnD-system Quantikine ELISA

The same Mtb antigen- and PHA-stimulated samples used for the Bio-Rad human 17-plex assay experiment 2, LINCO human 29-plex assay and RnD Systems fluorokine-(MAP) experiment 1 assay were also assessed by ELISA. The ELISA was done using the RnD Systems IFN-γ Quantikine ELISA kit (cat# DIF50) according to the manufacturer's instructions. Briefly, lyophilised Quantikine standard was reconstituted in distilled water and serially diluted one in two in kit standard diluent to produce a seven-point standard curve ranging from 15.6 pg/ml to 1000 pg/ml. Thereafter, 100 µl of assay diluent was added to the designated wells in a 96-well polystyrene microplate (provided with the kit) coated with polyclonal antibody against IFN-γ, followed immediately by 100 µl of standard, sample or control. The standard curve, samples and controls were run in duplicate. The plate was incubated for two hours at room temperature, washed and thereafter 200 µl of horseradish peroxydase (HRP)-conjugated IFN-γ antibody followed by 200 µl of substrate solution was added to the wells, followed by another incubation period and washing step between the two additions. After 30 minutes of incubation, 50 µl of stop solution was added to the wells and the plate read at 450 nm, with the wavelength correction set at 570 nm, on a multi-detection microplate reader (Bio-Tek instruments Inc, part # 7081000). Sample concentrations were determined using the KC4 microplate data analysis software, version 3.34, revision 12.

### Statistics: Manufacturer 1d, 2, 3 assays and ELISA comparison (Study 2)

The correlation between the concentrations of cytokines as measured by the different Immunoassays for the same sample was assessed by the mean of intra-class correlation coefficients and the Pearson product-moment correlation coefficient. The analysis was done using STATISTICA (version 7).

## Results

### Manufacturer 1 assay

#### Experiment 1

The recovery of the Bio-Rad human 17-plex was assessed using spiked whole blood culture supernatants from three healthy individuals and each of the supernatants was spiked at three different concentrations. Generally, a lack of accuracy was observed as illustrated in Table 1. At a high RP1 target, 21% of positive readings were in the recovery range of 70 to 130%, whereas only 12.4% were within that range when samples were read at a low RP1 target.

**Table 1:** Bio-Rad human 17-plex expected and observed cytokine concentrations and recovery (experiment 1).

	Expected (pg/ml)	High RP1 target		Low RP1 target	
		Observed (pg/ml)	Recovery (%)	Observed (pg/ml)	Recovery (%)
**IFN-gamma**	1797.5	OOR>	NA	9316	518.3
	179.7	600.25	334	491.23	273.36
	18.0	40.36	224.2	27.1	150
**TNF-alpha**	10351.4	8424.8	81.4	16605.5	160.4
	1035.1	2212	213.7	1582.7	152.9
	103.5	178.7	172.6	138.7	134.0
**IL-1beta**	3132.0	4082.4	130.3	10131.7	323.5
	313.2	1800.33	574.8	421.8	134.7
	31.3	56.6	180.7	49.4	157.7
**IL-2**	1762.7	1882.8	106.8	2895.7	164.3
	176.3	363.8	206.4	302.2	171.4
	17.6	40.3	228.7	32.6	185.0
**IL-4**	695.4	707.1	101.7	OOR>	NA
	69.5	202.8	291.6	164.7	236.8
	7.0	22.2	319.2	15.3	220.0
**IL-5**	5159.5	8582.8	166.3	13496	261.6
	516.0	2727.2	528.6	739.7	143.4
	51.6	96.4	186.8	79.5	154.1
**L-6**	3422.8	2817	82.3	8190.6	239.3
	342.3	893.2	261.0	550.6	160.9
	34.2	107.9	315.3	60.4	176.5
**IL-7**	5460.6	9190	168.3	10819.1	198.1
	546.1	1392	254.9	1032	189.0
	54.6	145.1	265.8	98.6	180.6
**IL-8**	3672.0	3702.8	100.8	5661.5	154.2
	367.2	861.3	234.6	613	166.9
	36.7	89	242.4	39.1	106.5
**IL-10**	4810.6	4540.7	94.4	7128.86	148.2
	481.1	1348.9	280.4	732.23	152.2
	48.1	98.43	204.6	75.53	157.0
**IL-12-p70**	4798.2	4641.6	96.7	OOR>	NA
	479.8	1191.7	248.4	942	196.3
	48.0	114	237.6	68.4	142.6
**IL-13**	762.5	1116.1	146.4	1401	183.7
	76.3	169.7	222.6	140.1	183.7
	7.6	15.7	205.8	14.1	184.8
**IL-17**	2765.6	3074.8	111.2	9773.8	353.4
	276.6	940.6	340.1	791.9	286.3
	27.7	110.2	398.4	70.6	255.2
**GM-CSF**	2552.7	4100.5	160.6	4716.7	184.8
	255.3	597.3	234.0	478.6	187.5
	25.5	55.1	215.8	42	164.5
**G-CSF**	2536.6	21494.94	847.4	3861	152.2
	253.7	564.8	222.7	431.6	170.1
	25.4	50.1	197.5	40.2	158.5
**MCP-1**	3412.6	5958.1	174.6	7755.5	227.3
	341.3	700.5	205.3	595.1	174.4
	34.1	16.7	48.9	7.5	22.0
**MIP-beta**	2544.7	1962.6	77.1	6157.1	242.0
	254.5	636.56	250.2	382.1	150.2
	25.4	47.5	186.7	44.9	176.4

Unstimulated whole blood culture supernatant samples were spiked at three different concentrations of recombinant cytokines. Samples were analysed on the Bio-plex system instrument and the recovery of each cytokine in the panel assessed. The expected concentrations, after subtraction of the endogenous levels of cytokines, are represented.

#### Experiment 2

In this study, recovery of the Bio-Rad human 17-plex was assessed for five different concentrations of individual cytokines. Fluorescence was read both at high and low RP1 targets, with 85 readings made for each RP1 target. A total of 65 readings out of 85 were positive for the low RP1 target, with 54% of these positive readings (41.2% of the total readings) falling within the acceptable recovery range of 70 to 130%. There were 62 positive readings out of 85 at the high RP1 target, with 61% of these (44.7% of the total readings) falling within the acceptable recovery range of 70 to 130%. The cytokines IFN-γ, TNF-α, IL-1β, IL-2, IL-4, IL-6, IL-7, IL-12p70, IL-13, IL-17, GM-CSF and MCP-1 measured most accurately when samples were read at the high RP1 target, whereas measurements of IL-5, IL-10, G-CSF and MIP-1β showed better recoveries when samples were read at a low RP1 target. Interfering interactions in the samples presumably led to falsely increased and signal inhibition for IL-8 detection, which resulted in out-of-range readings for four out of the five assessed concentrations.

**Figure 1:**
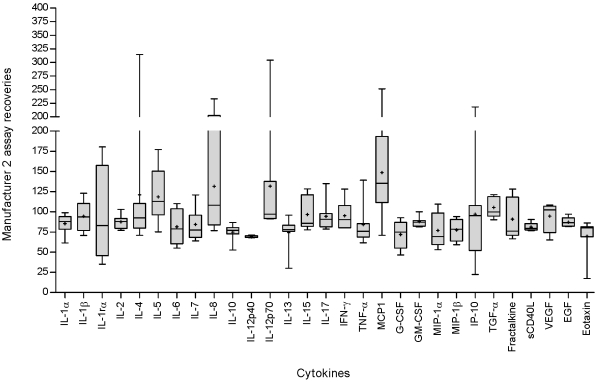
Recoveries of the Bio-Rad 17-plex assay. Un-stimulated whole blood culture supernatant samples from healthy donors were spiked at three (experiment 1) and five (experiment 2) different concentrations (2–8062 pg/ml) with the standard from the Bio-Rad 17-plex kit. Samples were assayed in duplicate and read at high and low RP1 targets on the Bio-plex system instrument. Recoveries were calculated for each of the cytokines in the panels for each of the spiked concentrations. The figure shows the recoveries obtained for each individual cytokine after two independent experiments.

The recoveries of cytokines included in the Bio-Rad human 17-plex panel are shown in Figure 1 (recoveries from two independent experiments). Detailed statistics on Bio-Rad human 17-plex recovery and variations are shown in Table 2.

**Table 2:** Detailed statistics of Bio-Rad 17-plex assay recoveries (two independent experiments).

Cytokines	IFN-g	TNF-a	IL-1b	IL-2	IL-4	IL-5	IL-6	IL-7	IL-8
Number of positive reads	14	13	13	13	13	13	13	13	10
Minimum	20.3	51.47	23.77	88.89	43.07	14.7	19.63	67.52	100.2
Median	98.37	91.43	114.5	106.3	101.7	115.7	92.05	168.3	104
Maximum	518.3	152.9	323.5	692.5	319.2	261.6	176.5	824	166.9
Mean	165.2	100.2	138.4	203.8	147	126.4	101.1	225	118.8
Std. Deviation	160.8	36.1	91.58	219.2	110.6	77.82	54.27	269.7	32.21
Std. Error	56.84	13.64	34.61	82.85	41.8	27.51	20.51	101.9	16.11
Coefficient of variation	97.32%	36.03%	66.15%	107.55%	75.22%	61.56%	53.67%	119.86%	27.13%

Cytokines	IL-10	IL-12p70	IL-13	IL-17	MCP1	MIP-1b	G-CSF	GM-CSF
Number of positive reads	14	14	13	13	12	12	13	13
Minimum	17.69	17.47	83.66	63.5	48.9	61.9	26.49	83.32
Median	86.81	96.84	146.4	111.2	69.05	93.07	105.5	160.6
Maximum	194.4	230.3	259.1	554.4	174.6	176.4	170.1	850.1
Mean	102.1	117.3	150.5	204.3	99.58	108.6	114.3	232.1
Std. Deviation	60.77	69.12	63.35	178.7	58.53	44.71	50.41	275.8
Std. Error	21.49	24.44	23.95	67.52	23.89	18.25	19.05	104.3
Coefficient of variation	59.50%	58.91%	42.09%	87.46%	58.77%	41.16%	44.11%	118.82%

Unstimulated whole blood culture supernatant samples were spiked at different concentrations with recombinant cytokines. Samples were analysed on the Bio-plex system instrument and the recovery of each cytokine in assessed after subtraction of the endogenous levels of cytokines.

### Manufacturer 2 assay

#### Experiment 1

The recovery of the cytokines forming part of the LINCO-Inc 29-plex panel were assessed at five different concentrations (5 000, 1 000, 500, 50 and 10 pg/ml), and read at high and low RP1 targets. The test showed an acceptable performance. A total of 145 readings were made at each RP1 target and 123 of these fell within the detection range when read at high RP1, compared to 136 out of 145 when read at the low RP1 target. Of the positive readings, 78.4% (66.2% of the total readings) read at the high RP1 target had recoveries falling within the acceptable range of 70 to 130%, whereas approximately 70% of positive readings (65.7% of the total readings) made at a low RP1 target achieved this acceptable recovery. Measurements of IFN-γ, IL-1β, IL-4, IL-6, IL-7, IP-10, MCP-1 and G-CSF were found to be most accurate when the plate was read at a high RP1 target, with recoveries falling between 70 to 130%, whereas those for TNF-α, IL-1α, IL-1rα, IL-2, IL-5, IL-10, IL-12p40, IL-12p70, IL-13, Fractalkine, MIP-1α, MIP-1β, GM-CSF, TGF-α, sCD40L, VEGF, Eotaxine and EGF were most accurate when read at a low RP1 target. IL-15 and IL-17 showed similar recoveries at both high and low RP1 targets. The level of background signal was very high for IL-8; this was most probably due to de fact that whole blood supernatant used for the spiking experiment was not pre-diluted. The coefficients of variation between the measurement at high RP1 and low RP1 targets were less than 5% when applicable (when both high and low RP1 showed positive readings).

#### Experiment 2

**Figure 2:**
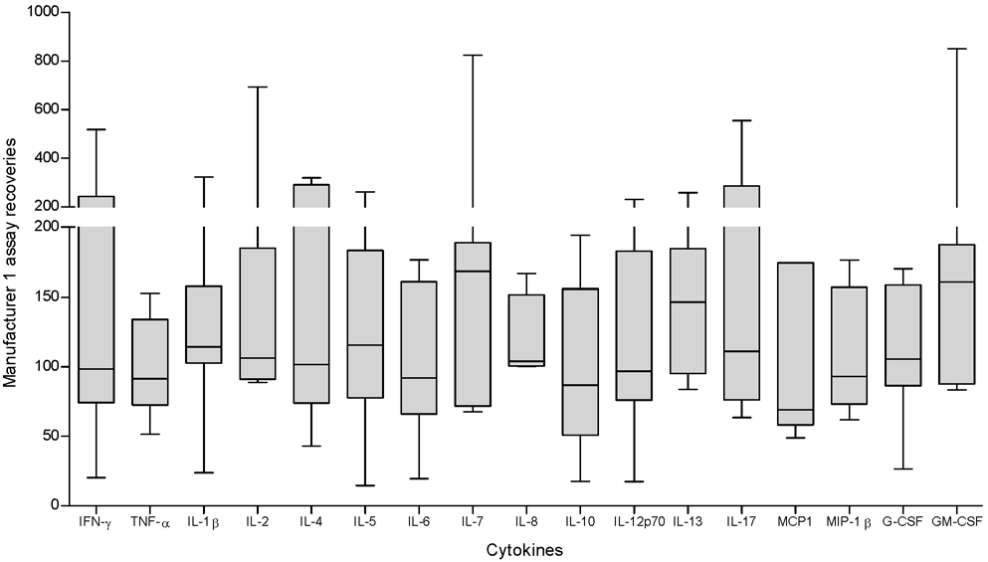
Recoveries of the Linco 29-plex assay. Un-stimulated whole blood culture supernatant samples from healthy donors were spiked at five (experiment 1) and two (experiment 2) different concentrations ranging from 10–5000 pg/ml with the standards from the LINCO 29-plex kit. Samples were assayed in duplicate and read at high and low RP1 targets on the Bio-plex system instrument. Recoveries were calculated for each of the cytokines in the panels for each of the spiked concentrations. The figure shows the recoveries obtained for each individual cytokine after two independent experiments.

The assessment of LINCO -plex kit was repeated only this time 21 cytokine were assessed. The repeat experiment was done using a healthy donor whole blood supernatant spiked at two different concentrations. The recoveries of all the cytokines in panel fell within the acceptable range of 70 to 130% except for MCP-1 and IL-6 for which the recoveries were 66 and 251% respectively. Figure 2 shows the recoveries of the LINCO -plex assay after the 2 independent experiments. Detail statistics are shown in Table 3.

**Table 3:** Detailed statistics of Linco-plex assay recoveries (two independent experiments).

Cytokines	IL-1α	IL-1β	IL-1rα (−)	IL-2	IL-4	IL-5	IL-6	IL-7
Number of positive reads	6	7	4	7	7	7	5	7
Minimum	61.44	70.7	35.02	77.09	70.92	75.23	55.12	64.04
Median	87.88	93.63	82.98	87.9	92.48	112.8	78.96	77.5
Maximum	99.01	123.2	180.5	102.9	314.4	177	110.2	120.9
Mean	85.43	94.49	95.37	87.58	121.1	118.6	81.48	84.32
Std. Deviation	12.92	18.51	61.23	8.522	86.17	34.65	22.5	19.67
Std. Error	5.275	6.997	30.62	3.221	32.57	13.09	10.06	7.433
Coefficient of variation	15.13%	19.59%	64.21%	9.73%	71.15%	29.22%	27.62%	23.32%

Cytokines	IL-8	IL-10	IL-12p40 (−)	IL-12p70	IL-13	IL-15	IL-17	IFN-g
Number of positive reads	4	7	4	7	7	7	7	7
Minimum	76.67	52.7	67.87	91	30.2	77.64	78.64	80.07
Median	108.1	77.15	68.75	97.1	78.02	85.7	90.58	90.29
Maximum	233	86.8	71	304.3	95.84	128.3	134.9	128.2
Mean	131.5	74.81	69.09	131.8	74.22	96.48	94.31	95.23
Std. Deviation	69.34	10.69	1.371	77.84	20.59	20.33	19.22	17.9
Std. Error	34.67	4.039	0.6857	29.42	7.781	7.685	7.264	6.767
Coefficient of variation	52.74%	14.28%	1.98%	59.08%	27.74%	21.07%	20.38%	18.80%

Cytokines	TNF-α	MCP1	G-CSF	GM-CSF	MIP-1α	MIP-1β (−)	IP-10	TGF-α (−)
Number of positive reads	7	6	5	7	5	4	7	5
Minimum	61.6	70.88	46.55	81.44	53.06	59.3	22.3	90.15
Median	76.02	135.3	74.83	87.1	69.19	78.55	95.25	99.96
Maximum	139.2	251.5	92.58	100.1	109.5	93.94	218.3	121.4
Mean	84.19	148.7	71.77	87.84	76.99	77.58	96.86	105.3
Std. Deviation	25.56	60.57	17.57	6.146	21.91	14.22	61.94	12.94
Std. Error	9.659	24.73	7.856	2.323	9.8	7.109	23.41	5.785
Coefficient of variation	30.35%	40.73%	24.47%	7.00%	28.46%	18.33%	63.94%	12.28%

Cytokines	Fractalkine (−)	sCD40L (−)	VEGF (−)	EGF (−)	Eotaxin
Number of positive reads	5	5	4	5	7
Minimum	66.46	76.8	65.1	81.6	17.3
Median	76.12	79.4	102.4	86.58	80.17
Maximum	128.2	90.38	108.3	97.24	86.1
Mean	90.79	81	94.53	87.18	69.37
Std. Deviation	26.22	5.407	19.82	6.149	23.74
Std. Error	11.72	2.418	9.911	2.75	8.973
Coefficient of variation	28.88%	6.67%	20.97%	7.05%	34.23%

**(−)** Cytokines not included in the second test.

Unstimulated whole blood culture supernatant samples were spiked at different concentrations with recombinant cytokines. Samples were analysed on the Bio-plex system instrument and the recovery of each cytokine assessed after subtraction of the endogenous levels of cytokines.

The reproducibility of the LINCO kits was done by measuring cytokine concentrations of the same whole blood supernatants across five LINCO 29-plex kits. Data analysis showed that the coefficient of variation, standard deviation and error were within acceptable ranges for most of the cytokines (see Table 4 for more details).

**Table 4:** Linco 29-plex assay reproducibility.

Cytokines	IL-1B	IL-2	IL-1ra	IL-4	IL-5	EGF	IL-6	IL-7	TGF−α
Number of experiments	5	5	5	5	5	5	5	5	5
Minimum concentration	6.46	1.6	52.92	5.8	6.05	1.6	238.7	3.53	1.6
Median concentration	7.27	1.6	59.7	9.36	6.87	1.6	258.8	3.99	1.6
Maximum concentration	9.17	4.33	79.18	14.09	8.55	1.6	304.9	8.69	1.6
Mean concentration	7.378	2.538	65.58	10.29	7.038	1.6	261.6	5.182	1.6
Std. Deviation	1.099	1.313	12.37	3.294	0.9504	0	26.13	2.19	0
Std. Error	0.4914	0.5872	5.533	1.473	0.425	0	11.69	0.9794	0
Coefficient of variation	14.89%	51.73%	18.87%	32.01%	13.50%	0.00%	9.99%	42.26%	0.00%

Cytokines	Fractalkine	IL-8	IL-10	IL-12p70	IL-13	IL-15	IL-17	IL-1α	IFN-γ
Number of experiments	5	5	5	5	5	5	5	5	5
Minimum concentration	1.6	828.4	143.1	1.6	18.9	1.6	12.34	22.73	687.4
Median concentration	1.6	960.2	161.8	1.6	20.15	1.6	13.86	24.16	801.5
Maximum concentration	43.48	1153	200.4	4.71	26.7	1.6	20.83	32.38	993.7
Mean concentration	11.31	969.8	167.9	2.222	21.17	1.6	14.96	25.65	807.1
Std. Deviation	18.21	116.9	21.57	1.391	3.238	0	3.351	3.851	127.1
Std. Error	8.145	52.29	9.648	0.622	1.448	0	1.499	1.722	56.85
Coefficient of variation	161.01%	12.06%	12.85%	62.59%	15.29%	0.00%	22.41%	15.01%	15.75%

Cytokines	G-CSF	GM-CSF	TNF−α	Eotaxin	MCP-1	sCD40L	IL-12p40	MIP-1α	MIP-1β
Number of experiments	5	5	5	4	5	5	5	5	5
Minimum concentration	22.19	52.82	33.06	2.52	430.2	9	10.2	604.5	466.3
Median concentration	30.91	58.65	36.34	7.965	520.4	10.05	22.11	665.6	516.1
Maximum concentration	34.29	70.71	49.75	18.78	559	18.53	38.45	777.7	623.4
Mean concentration	29.65	59.71	38.3	9.308	502.6	12.85	21.96	690.1	519.2
Std. Deviation	4.656	6.728	6.618	7.101	50.63	4.788	10.45	75.02	64.1
Std. Error	2.082	3.009	2.96	3.55	22.64	2.141	4.674	33.55	28.67
Coefficient of variation	15.70%	11.27%	17.28%	76.29%	10.07%	37.26%	47.58%	10.87%	12.35%

Cytokine	IP-10	VEGF
Number of experiments	5	5
Minimum concentration	140	30.58
Median concentration	159.1	52.07
Maximum concentration	287.8	64.79
Mean concentration	182.1	50.43
Std. Deviation	60.02	13.84
Std. Error	26.84	6.189
Coefficient of variation	32.96%	27.44%

Five aliquots of the same PHA-stimulated whole blood supernatant were produced and each aliquot was run on different plates and on different days.

### Manufacturer 3 assay

#### Experiment 1

The recoveries of 13 cytokines measured in whole blood culture supernatant and serum samples were assessed for six different concentrations using the RnD Systems Fluorokine-MAP assay. A total of 78 readings were made using whole blood culture supernatant and 67 from serum samples. All whole blood supernatant and serum sample readings were positive and within the standard curve range. A total of 67% of whole blood supernatant samples achieved recoveries within 70 to 130%, compared to approximately 56% of the serum samples.

#### Experiment 2

**Figure 3:**
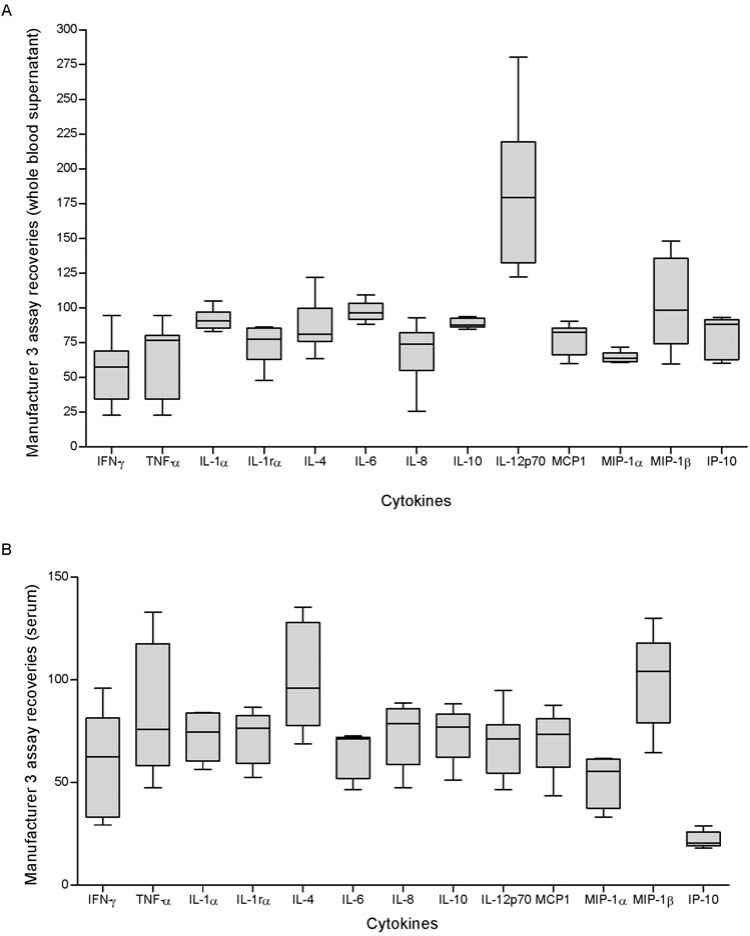
Recoveries of RnD System's Fluorokine-MAP 13-plex base kits A and B. (A) Un-stimulated whole blood culture supernatant samples from a healthy donor were spiked at different concentrations (14–19000 pg/ml) with the standards from RnD-System Fluorokine-MAP base kits A and B. Samples were assayed in duplicate, read at a low RP1 target on the Bio-plex system instrument and recoveries calculated for each of the cytokines in the panel. The figure shows the individual cytokine's best recovery obtained. (B) Serum samples from a healthy donor were spiked at concentrations (43.8–19000 pg/ml) with the standards from RnD-System's Fluorokine-MAP base kits A and B. Samples were assayed in duplicate, read at a low RP1 target on the Bio-plex system instrument and recoveries calculated for each of the cytokines in the panel. The figure shows the individual cytokine's recoveries obtained. The experiment was repeated for IFN-γ, ΤΝF-α and IL-4 only.

The recoveries of IFN-γ, TNF-α and IL-4 were assessed for seven different concentrations in whole blood culture supernatant and serum samples. In this experiment all whole blood supernatant readings were positive, whereas the detection limits in spiked serum samples were 44 pg/ml. About 50% of spiked whole blood supernatant achieved acceptable recovery (70 to 130%) compared to 75% for serum samples that were within detection range. Details on cytokine recoveries after the two independent experiments are shown in Figure 3 detailed statistics in Table 5.

**Table 5:** RnD System's Fluorokine-MAP kits assay recoveries detailed statistics (two independent experiments).

Whole blood Supernatant								
Cytokines	IFN-γ (*)	TNF-α (*)	IL-1α	IL-1rα	IL-4 (*)	IL-6	IL-8	IL-10
Number of positive reads	13	13	6	6	13	6	6	6
Minimum	22.73	22.73	82.99	47.73	63.66	88.34	25.6	84.6
Median	57.55	76.71	90.76	77.52	81.01	96.28	74.01	87.68
Maximum	94.36	94.36	104.9	86.32	121.8	109.2	92.93	93.5
Mean	54.86	63.31	91.64	73.72	86.62	97.41	68.23	88.75
Std. Deviation	21.9	25.14	7.599	14.38	17.7	7.541	22.86	3.453
Std. Error	6.074	6.974	3.102	5.87	4.909	3.079	9.333	1.41
Coefficient of variation	39.92%	39.72%	8.29%	19.50%	20.43%	7.74%	33.51%	3.89%

Cytokines	IL-12p70	MCP1	MIP-1α	MIP-1β	IP-10
Number of positive reads	6	6	6	6	6
Minimum	122.1	59.9	60.69	59.57	60.14
Median	179.4	82.48	63.92	98.28	88.24
Maximum	280.2	90.39	71.71	148	93.05
Mean	182.6	77.91	64.67	102.4	80.71
Std. Deviation	56.27	11.38	4.02	32.9	14.74
Std. Error	22.97	4.645	1.641	13.43	6.019
Coefficient of variation	30.81%	14.60%	6.22%	32.12%	18.27%

Serum								
Cytokines	IFN-γ (*)	TNF-α (*)	IL-1α	IL-1rα	IL-4 (*)	IL-6	IL-8	IL-10
Number of positive reads	10	9	5	5	9	5	5	5
Minimum	29.33	47.49	56.34	52.55	68.86	46.58	47.59	51.28
Median	62.64	75.86	74.51	76.46	95.91	71.15	78.61	76.97
Maximum	95.91	133	84.16	86.62	135.3	72.72	88.71	88.34
Mean	61.04	87.51	72.65	72.07	101.2	63.83	73.62	73.63
Std. Deviation	23.87	31.61	12.18	13.13	25.39	11.53	16.08	13.68
Std. Error	7.549	10.54	5.447	5.87	8.462	5.155	7.192	6.116
Coefficient of variation	39.11%	36.12%	16.77%	18.21%	25.08%	18.06%	21.84%	18.57%

Cytokines	IL-12p70	MCP1	MIP-1α	MIP-1β	IP-10
Number of positive reads	6	5	5	5	6
Minimum	46.58	43.56	33.23	64.53	18.04
Median	71.3	73.44	55.49	104.2	20.51
Maximum	94.84	87.65	61.87	130	28.8
Mean	69	70.12	50.66	99.63	22.02
Std. Deviation	16.33	16.17	12.62	23.74	4.018
Std. Error	6.665	7.231	5.645	10.62	1.641
Coefficient of variation	23.66%	23.06%	24.92%	23.83%	18.25%

(*) Cytokines included in both experiment 1 and 2.

Unstimulated whole blood culture supernatant and serum samples were spiked at different concentrations with recombinant cytokines. After samples were measured and the level endogenous levels of cytokines subtracted the recovery of each cytokine was assessed. Only IFN-γ, ΤΝF-α and IL-4 were included in the second experiment.

### RnD System's ELISA

The RnD System's IFN-γ ELISA was used as a comparative test against which the different luminex kits where compared. Samples tested included antigen-stimulated samples with their negative and positive controls. As expected, the negative control showed a very low level of IFN-γ, whereas the positive control and antigen-stimulated samples showed higher levels of IFN- γ (Table 6).

**Table 6:** IFN-γ-based comparison of ELISA, LINCO 29-plex, Bio-Rad 17-plex and RnD Systems Fluorokine-MAP-13-plex assays.

	RnD Systems ELISA (pg/ml)	LINCO 29-plex (pg/ml)	Bio-Rad 17-plex (pg/ml)	RnD Systems Fluorokine-MAP (pg/ml)
**Whole blood supernatant**				
**PHA-stimulated**	1731.38	451.56	64.7	86.83
**Negative Control**	0	1.39	0	NA
**Mtb derived antigen 1 (supernatant A)**	1181.6	969.12	0	NA
**Mtb derived antigen 1 (supernatant B)**	908.83	950.62	2.14	NA
**Mtb derived antigen 2 (supernatant B)**	839.42	536.92	193.53	NA
**Mtb derived antigen 3 (supernatant D)**	489.1	247	49.08	30.26
**Mtb derived antigen 2 (supernatant C)**	235.67	125.97	104.94	17.65
**Mtb derived antigen 4 (supernatant E)**	118.9	133.82	172.19	NA
**Mtb derived antigen 3 (supernatant C)**	57.5	55.63	0	1.56
**Mtb derived antigen 5 (supernatant E)**	23.78	0.44	99.2	0.28

NA: Not applicable because the sample was not measured with the RnD Systems Fluorokine-MAP base kit A

Supernatant was generated using whole blood from a healthy donor (A-E) in six day assays stimulated with 5 differents Mtb derived antigens and with phytohaemagglutinin (PHA). The LINCO 29-plex measurement most closely resembled that of the RnD Systems ELISA in absolute value, and followed a similar trend, but absolute values for all three luminex assay fell below that of the ELISA.

### Manufacturer 1, 2, 3 luminex assays and RnD Systems ELISA: an IFN-γ based comparison

Poor correlations were observed between the Bio-Rad luminex assay and the other assays. The correlation between the Bio-Rad luminex kit and the RnD Systems ELISA measurement gave an ICC of agreement of −0.01 and a Pearson correlation coefficient (r) of −0.09. The intra-class correlation coefficients (ICC) of agreement and the Pearson product-moment correlation coefficient (r) between LINCO luminex kits and RnD Systems ELISA kits were for the first test 0.64 (ICC) and 0.75 (r) and for the second test 0.75 and (ICC) and 0.84 (r) suggesting a posive correlation between LINCO luminex kits and ELISA. The correlation analysis between RnD Systems Fluorokine-MAP and RnD Systems ELISA was also shown to be positive with an ICC of agreement of 0.1 and a Pearson correlation coefficient (r) of 0.99. The correlation between the different luminex kits measurements for the cytokines present in the three kit panels is shown in Table 7.

**Table 7:** Correlation between ELISA, LINCO 29-plex, Bio-Rad 17-plex and RnD Systems Fluorokine-MAP 13-plex assays.

	Rater 1	Rater 2	ICC agreement	ICC consistency	r
**IFN-γ**	RnD System ELISA	LINCO 29-plex	0.638	0.67	0.75
	RnD System ELISA	Bio-Rad 17-plex	−0.014	−0.02	−0.1
	RnD System ELISA	RnD-System MAP	0.1	0.1	0.99
	LINCO 29-plex	Bio-Rad 17-plex	−0.06	−0.085	−0.2
	LINCO 29-plex	RnD-System MAP	0.26	0.36	0.97
	Bio-Rad 17-plex	RnD-System MAP	0.18	0.21	0.22
**IL-2**	LINCO 29-plex	Bio-Rad 17-plex	0.56	0.65	0.71
**IL-4**	LINCO 29-plex	Bio-Rad 17-plex	0.098	0.17	0.23
	LINCO 29-plex	RnD-System MAP	−0.005	−0.01	−0.3
	Bio-Rad 17-plex	RnD-System MAP	0.002	0.0064	0.23
**IL-6**	LINCO 29-plex	Bio-Rad 17-plex	−0.05	−0.07	−0.1
	LINCO 29-plex	RnD-System MAP	0.77	0.74	0.88
	Bio-Rad 17-plex	RnD-System MAP	−0.08	−0.075	−0.1
**IL-8**	LINCO 29-plex	Bio-Rad 17-plex	0.017	0.03	0.04
	LINCO 29-plex	RnD-System MAP	0.3	0.56	0.73
	Bio-Rad 17-plex	RnD-System MAP	−0.046	−0.042	−004
**IL-10**	LINCO 29-plex	Bio-Rad 17-plex	−0.02	−0.02	−004
	LINCO 29-plex	RnD-System MAP	0.007	0.014	0.68
	Bio-Rad 17-plex	RnD-System MAP	0.004	0.007	0.45
**GM-CFS**	LINCO 29-plex	Bio-Rad 17-plex	−0.014	−0.021	−0.2
**TNF-α**	LINCO 29-plex	Bio-Rad 17-plex	−0.06	−0.067	−0.1
	LINCO 29-plex	RnD-System MAP	0.9	0.9	0.91
	Bio-Rad 17-plex	RnD-System MAP	0.42	0.38	0.38
**IL-1β**	LINCO 29-plex	Bio-Rad 17-plex	−0.27	−0.27	−0.3
**IL-5**	LINCO 29-plex	Bio-Rad 17-plex	−0.1	−0.093	−0.1
	LINCO 29-plex	RnD-System MAP	−0.046	−0.038	−0.1
	Bio-Rad 17-plex	RnD-System MAP	−0.18	−0.15	−0.5
**IL-13**	LINCO 29-plex	Bio-Rad 17-plex	−0.14	−0.138	−0.2
**IL-17**	LINCO 29-plex	Bio-Rad 17-plex	−0.1	−0.11	−0.2
**MCP-1 (MCAF)**	LINCO 29-plex	Bio-Rad 17-plex	0.5	0.48	0.48
	LINCO 29-plex	RnD-System MAP	0.02	0.043	0.62
	Bio-Rad 17-plex	RnD-System MAP	−0.008	−0.018	−0.2
**MIP-1β**	LINCO 29-plex	Bio-Rad 17-plex	0.04	0.037	0.05
	LINCO 29-plex	RnD-System MAP	0.25	0.25	0.73
	Bio-Rad 17-plex	RnD-System MAP	0.27	0.26	0.43
**MIP-1α**	LINCO 29-plex	RnD-System MAP	0.72	0.78	0.95
**IL-7**	LINCO 29-plex	Bio-Rad 17-plex	0.11	0.25	0.36
**IL-12**	LINCO 29-plex	Bio-Rad 17-plex	−0.16	−0.14	−0.1
**G-CSF**	LINCO 29-plex	Bio-Rad 17-plex	0.004	0.005	0.01
**IL1-α**	LINCO 29-plex	RnD-System MAP	0.33	0.49	0.97
**IL-1rα**	LINCO 29-plex	RnD-System MAP	0.23	0.39	0.85

10 Whole blood supernatant from a healthy donor were stimulated with antigens in six day assays. The table shows the intra-class correlation coefficients (ICC) of agreement and consistency as well as Pearson correlation coefficients (r) between measurements obtained with the different luminex kits and the ELISA.

## Discussion

This validation study evaluated three commercially available cytokine multiplex bead immunoassays from Bio-Rad, LINCO-Inc and RnD Systems. The results suggest that, for the particular samples tested in this study, the LINCO Inc human 29-plex and the RnD Systems Fluorokine-MAP assays were the most accurate for the measurement of cytokine concentrations in whole blood culture supernatant and achieved good recovery ranges and reproducibility for most cytokines whereas the performance of the Bio-Rad human 17-plex assay was suboptimal.

The comparative study, including the Bio-Rad human 17-plex assay, LINCO 29-plex assay, RnD Systems Fluorokine-MAP assay and RnD Systems ELISA, was made based on IFN-γ responses in antigen-stimulated whole blood culture supernatant. It was found that all assays were capable of differentiating the positive and negative controls. Moreover, they were able to efficiently pick up the antigen-specific IFN-γ responses when applicable, with the exception of the Bio-Rad human 17-plex assay, where IFN-γ levels in two of the antigen-stimulated samples (ESAT-CFP-10 and Rv1115) went undetected.

Concentrations of IFN-γ measured by the LINCO 29-plex assay, RnD Systems Fluorokine-MAP assay and ELISA correlated, but results obtained using the Bio-plex assay correlated poorly with values obtained using the other three assays. Very similar to the findings by DuPont *et al*. [5], a very strong correlation between the level of IFN-γ measured by ELISA and the LINCO-plex kit in whole blood culture supernatant was found in the present study. Furthermore LINCO-plex assay, RnD Systems Fluorokine-MAP assay and the Bio-Rad 17-plex correlation of 13 cytokines showed a positive correlation between LINCO 29-plex and the RnD Systems Fluorokine-MAP assay for most of the cytokines, whereas the Bio-Rad 17-plex assay correlations to LINCO 29-plex assay and the RnD Systems Fluorokine-MAP assay were frequently negative. This contradicts a study by Khan *et al*. [11], who showed that cytokine levels of IL-6, IL-8, and TFN-α measured by the Bio-Rad Bio-plex assay have similar trends to the LINCO-plex and RnD Systems Fluorokine-MAP measurements at least for IL-6, IL-8, and TFN-α.

Although recoveries should ideally not fall outside the acceptable range, measurements may be considered useable if the recovery remains constant across different sample types and dilutions. This was not the case when measuring cytokines using the Bio-Rad kits in our study. Therefore it would be impossible to compensate for any inaccuracy evident in any one sample matrix. Discrepancies observed using the Bio-plex kits may be partly explained by the presence of interfering proteins such as heterophilic antibodies [10], [14]. De Jager *et al*. [3] have described methods to avoid heterophilic antibody interference in plasma and synovial fluid that improved the performance of the multiplex immunoassay. However, any manipulation of samples in clinical studies is not necessarily advantageous due to possible unforeseen effects on the results.

It may therefore be necessary to perform careful optimisation and validation of any commercial multiplex cytokine assays prior to large-scale clinical studies, as the quality controls supplied with the kits to measure standard curve integrity can only guarantee the accuracy or sensitivity of the assay if they are reconstituted and measured in the same matrix type as the samples investigated. Matrix effects appear to play a major role in assay performance and the type of sample tested may therefore have serious effects on assay performance. It is clear that, at the present moment, the theoretical capabilities of this new technology cannot be fully achieved in practice [12]. Researchers using these kits should include replicates of samples as well as negative and positive (low, medium and high) controls with known concentrations of the cytokines of interest to aid in them in interpretation of results. Furthermore, controls should be included that reflect both the diluents used to reconstitute the standard supplied with the kit and the sample matrix tested in order to account for possible matrix effects. This will allow the assessment of linearity and recovery and aid in the choice of best standard curve regression and optimal calibration [2].

In conclusion, the most appropriate use for multiplex cytokine assays based on luminex technology currently is as a screening tool, for example for the selection of candidate markers characteristic of disease-associated immune responses. Promising candidates can then be validated using a method with higher accuracy and proven reliability, such as ELISA.
